# AFFECTIONATE PHYSICAL TOUCH MITIGATES PAIN AND EMOTIONAL DISTRESS IN OLDER ADULTS

**DOI:** 10.1093/geroni/igz038.2984

**Published:** 2019-11-08

**Authors:** Jinshil Hyun, Richard B Lipton, Ruixue E Zhaoyang, Jennifer E Graham-Engeland, Jelena M Pavlovic, Martin J Sliwinski

**Affiliations:** 1 Albert Einstein College of Medicine, Bronx, New York, United States; 2 The Pennsylvania State University, University Park, Pennsylvania, United States

## Abstract

Although research suggests that social interactions can decrease pain and emotional distress, it is unclear what produces these salubrious effects. We examined whether older adults experienced lower pain and emotional distress after two types of social interactions (affectionate physical contact and non-physical pleasant interactions) using data from the Einstein Aging Study (N=193, age=70-92). Participants completed a 14-day ecological momentary assessment protocol via which they reported the quality of recent social interaction, types of physical touch, levels of current stress, negative affect, and pain intensity five times a day. Multilevel models indicated that, following affectionate physical contact, individuals reported low levels of current pain intensity, negative affect, and stress (ps<.05). Following a pleasant non-physical interaction, individuals reported low negative affect (p<.05); pleasant interactions did not predict current pain or stress. Results highlight the potential unique utility of affectionate physical contact versus mere pleasant social interactions in older adults’ daily lives.

